# Impact of cycling and walking on adiposity and healthcare costs among adults: longitudinal study

**DOI:** 10.1590/0102-311XEN102623

**Published:** 2024-02-26

**Authors:** Rafael Orbolato, Rômulo Araújo Fernandes, Bruna Camilo Turi-Lynch, Monique Yndawe Castanho Araujo, Izabela dos Santos Ferro, Luis Alberto Gobbo, Everton Alex Carvalho Zanuto, Jamile Sanches Codogno

**Affiliations:** 1 Universidade Estadual Paulista Júlio de Mesquita Filho, Presidente Prudente, Brasil.; 2 Lander University, Greenwood, U. S. A.; 3 Faculdades de Dracena, Fundação Dracenense de Educação e Cultura, Dracena, Brasil.

**Keywords:** Exercise, Body Mass Index, Health Care Costs, Exercício Físico, Índice de Massa Corporal, Custos de Cuidados de Saúde, Ejercicio Físico, Índice de Masa Corporal, Costos de Atención en Salud

## Abstract

Leisure-time physical activity seems relevant to prevent the development of chronic diseases and obesity. However, not much is known about the economic burden of these healthy behaviors, mainly in longitudinal designs. This study aimed to analyze the impact of walking and cycling on leisure-time on adiposity and healthcare costs among adults. This longitudinal study was conducted at a medium-size Brazilian city and included 198 participants with no missing data attended in the Brazilian Unified National Health System. Cycling and walking were assessed by a questionnaire with a face-to-face interview at four time-points (baseline, 6-month, 12-month, and 18-month). Healthcare costs were assessed using medical records. Adiposity markers included waist circumference and body fatness. Over the follow-up period, participants who were more engaged in cycling presented lower body fatness (p-value = 0.028) and healthcare costs (p-value = 0.038). However, in the multivariate model, the impact of cycling on costs was not significant (p-value = 0.507) due to the impact of number of chronic diseases (p-value = 0.001). Cycling on leisure-time is inversely related to adiposity in adults, whereas its role on preventing chronic diseases seems the main pathway linking it to cost mitigation.

## Resumo

A atividade física no lazer parece relevante para prevenir o desenvolvimento de doenças crônicas e obesidade. No entanto, pouco se sabe sobre o impacto econômico destes comportamentos saudáveis, principalmente em estudos longitudinais. O objetivo deste estudo foi analisar o impacto da caminhada e do ciclismo como atividades de lazer na adiposidade e nos custos de saúde em adultos. Este estudo longitudinal foi realizado em uma cidade brasileira de médio porte e incluiu 198 participantes sem dados indisponíveis atendidos no Sistema Único de Saúde brasileiro. A caminhada e o ciclismo foram avaliados por meio de questionário e entrevista presencial em quatro momentos (linha de base, 6 meses, 12 meses e 18 meses). Os custos de saúde foram avaliados por meio de prontuários médicos. Os marcadores de adiposidade incluíram circunferência da cintura e gordura corporal. Durante o período de acompanhamento, os participantes que praticavam mais ciclismo apresentaram menos gordura corporal (p = 0,028) e custos de saúde (p = 0,038). Porém, no modelo multivariado, o impacto do ciclismo nos custos deixou de ser significativo (p = 0,507) devido ao impacto do número de doenças crônicas (p = 0,001). O ciclismo no momento de lazer está inversamente relacionado à adiposidade em adultos, enquanto o seu papel na prevenção de doenças crônicas parece ser o principal aspecto que o liga à redução de custos.

## Resumen

La actividad física en el ocio parece relevante para prevenir el desarrollo de enfermedades crónicas y la obesidad. Sin embargo, poco se sabe sobre el impacto económico de estos comportamientos saludables, especialmente en estudios longitudinales. El objetivo de este estudio fue analizar el impacto de caminar y andar en bicicleta como actividades de ocio sobre la adiposidad y los costos de salud en adultos. Este estudio longitudinal se llevó a cabo en una ciudad brasileña de tamaño mediano e incluyó a 198 participantes sin datos indisponibles atendidos en el Sistema Único de Salud brasileño. Se evaluaron los hábitos de caminar y andar en bicicleta mediante un cuestionario y una entrevista cara a cara en cuatro momentos (inicial, 6 meses, 12 meses y 18 meses). Los costos de atención médica se evaluaron utilizando registros médicos. Los marcadores de adiposidad incluyeron la circunferencia de la cintura y la grasa corporal. Durante el período de seguimiento, los participantes que practicaban más ciclismo presentaron menos grasa corporal (p = 0,028) y costos de salud (p = 0,038). Sin embargo, en el modelo multivariado, el impacto del ciclismo en los costos dejó de ser significativo (p = 0,507) debido al impacto del número de enfermedades crónicas (p = 0,001). El hábito de andar en bicicleta en los momentos de ocio está inversamente relacionado con la adiposidad en los adultos, mientras que su papel en la prevención de enfermedades crónicas parece ser el principal aspecto que lo vincula con la reducción de costos.

## Introduction

The high prevalence of insufficient physical activity constitutes a worldwide public health problem, which has significantly increased among adults ^1^ ^,^ ^2^. Insufficient physical activity is particularly harmful to human health due to its relevant role in the development of obesity and chronic diseases ^3^ ^,^ ^4^. Physical inactivity and sedentary behavior also increase the risk of premature mortality ^5^, whereas the regular engagement in good physical activity practice decreases mortality risk, regardless of chronic diseases ^6^. Therefore, this background ratifies the relevance of public health programs targeting the promotion of physical activity among adults.

Regarding physical activity promotion, leisure-time physical activity seems to be more relevant than other domains to prevent the development of chronic diseases and obesity. Regarding this domain, walking and cycling are important manifestations of physical exercise among adults ^7^, being potential behaviors to prevent chronic diseases and hence mitigate health costs. However, in developing nations, not much is known about the economic burden of walking and cycling ^8^ ^,^ ^9^.

Consistent information supports the idea that building walking and cycling track networks is beneficial to society in terms of air pollution, public transport, and health population ^10^. For healthcare costs, cross-sectional studies have described an inverse relationship between engagement in walking and lower healthcare costs ^8^ ^,^ ^11^ ^,^ ^12^ ^,^ ^13^, whereas few studies address the economic impact attributed to cycling ^14^. Although walking and cycling are common and accessible forms of transportation among adults ^7^ ^,^ ^15^, aging leads to reduction in both behaviors, while increasing chronic diseases occurrence and healthcare costs ^4^ ^,^ ^15^ ^,^ ^16^, especially after the fifth decade of life ^4^ ^,^ ^17^, placing this portion of the population at increased risk and raising questions on how walking and cycling could be beneficial in reducing costs.

Moreover, most data describing the association of walking and cycling with economic aspects are based on cross-sectional surveys and carried out in developed settings ^8^ ^,^ ^9^, leading to technical questions about its application in developing nations. Such issue seems even more relevant in the Brazilian scenario, in which healthcare assistance is financed by public fundings (at all levels of complexity).

Therefore, this study aimed to analyze, in a longitudinal design, the impact of walking and cycling on leisure-time on adiposity and healthcare costs among adults aged 50 years or older.

## Methods

### Sample and sampling

The sample was composed of adults aged 50 years or older who were selected in two basic health units (BHU) in two different geographical regions of the metropolitan area of Presidente Prudente (medium size municipality, with 220,000 inhabitants), Western São Paulo State, Brazil, and with Human Development Index (HDI) of 0.806. Sample size estimation considered a minimum difference for healthcare costs between sufficiently active (standard deviation [SD] = USD 22.86) and insufficiently active patients (SD = USD 15.03) of USD 6.27 ^18^, according to previous methodology ^19^, 80% statistical power, and 5% alpha error (Z = 1.96). The minimum sample size was estimated in 75 participants per group (n = 75 higher engagement in cycling/walking, and n = 75 lower engagement in cycling/walking).

The Brazilian Unified National Health System (SUS) offers primary, secondary, and tertiary healthcare services to all population free of charge. The BHU are the most distal arm of SUS since every single Brazilian municipality has at least one BHU (small to medium facilities located in different regions of the city), offering a large variety of primary care services (e.g., medical consultations [different specialties], vaccination, dental care, physical therapy). Municipal Health Department pointed out the two BHU that held our longitudinal study, which were selected due to the high number of people attended.

The same research team contacted and took measurements (face-to-face interview, anthropometric measurements, and bioelectrical impedance analysis - BIA) of all participants at the BHU (the research team was composed of previously trained PhD, MSc, and undergraduate students of the physical education and physical therapy programs, as well as the researcher responsible by the study). The fieldwork lasted 30 days in each BHU, in which all patients with a scheduled appointment were considered eligible for the study. Participants were contacted and the research team checked all inclusion criteria, as follows: age ≥ 50 years old and at least one medical consultation scheduled in the BHU in the six months before the first contact.

All patients who agreed to participate and fulfilled all inclusion criteria were followed for 18 months and assessed every six months (baseline [March/2014], 6-month [September/2014], 12-month [March/2015], and 18-month [September/2015]). In all stages of the study, the researchers repeated the face-to-face interview, anthropometric measurements, and BIA. At baseline, the sample comprised 327 participants, but at the end of the 18-month follow-up, the final sample comprised 198 participants with no missing data. Regarding how the final sample was representative of the general Brazilian adult population, 82% of Brazilian adults are estimated to live in urban areas (like our sample) and, regardless of dwelling area, 78% of adults do not have access to private health insurance, leading them to search exclusively for healthcare services offered by SUS ^17^. Moreover, similar to the general Brazilian adult population, our sample had a higher number of women than men, and approximately 40% of adults were illiterate or had incomplete elementary education ^17^.

The Institutional Review Board of the São Paulo University previously approved the study protocol (n. 241,291/2013). At baseline, all participants signed a written consent form agreeing to participate in the follow-up study.

### Dependent variable: healthcare costs

Direct healthcare costs were computed from the perspective of SUS in a time horizon of 18 months considering a bottom-up approach (focused on individual costs) ^20^. Healthcare costs were assessed using all procedures registered in the patients’ medical records (e.g., medical consultations [all specialties], medicine released, tests, physical therapy consultations) following previously validated methods ^8^ ^,^ ^15^ ^,^ ^18^. Additionally, all public expenditures related to the maintenance of the BHU (nurses’ wages, telephone, electric, and water bills of the BHU) were registered during the 18 months. All medical procedures in the last six months before the baseline were considered, as well as medical procedures registered during the 18-month follow-up, accounting for 24 months of healthcare costs. Regarding the period to observe real differences in healthcare costs, previous data identify that a minimum of 12 months of follow-up is sufficient to capture the potential impact of physical activity on healthcare costs among adults ^11^ ^,^ ^14^.

To convert all these medical procedures and maintenance expenditures into currency (Brazilian currency, BRL), the Municipal Health Department provided all values paid for all procedures and services above described ^11^ ^,^ ^17^ ^,^ ^18^. Costs were updated in accordance with the official Brazilian inflation index (Extended National Consumer Price Index - IPCA) ^20^, from the date data was obtained until December 2022, and converted into USD using the official exchange rate of the same date (USD exchange rate at 5.21), published by the Brazilian Central Bank. The variable denoting all the healthcare costs accumulated by each participant during the follow-up period was named “Healthcare costs 24 months”.

### Dependent variable: anthropometry and bioelectrical impedance analysis

Body composition was assessed using the BIA (InBody, model 230, https://inbody.com/en) at baseline and follow-up periods. All assessments happened during the morning, respecting 24h without physical exercise, no caffeine consumption (coffee), and an empty bladder. The software provided by the manufacturer provided data about body fatness percentage (%BF). Body mass index (BMI [kg/m^2^]) was calculated using body weight (electronic scale [maximum 150kg]) and height (wall mounted stadiometer [maximum 200cm]). Waist circumference (WC - metallic tape [cm]) was used as a proxy for abdominal obesity.

### Independent variable: walking and cycling

Physical activity was assessed with face-to-face interviews, using a validated questionnaire translated into Brazilian Portuguese ^21^ ^,^ ^22^. The questionnaire comprises 16 questions with Likert scale answers, and covers three physical activity domains: occupational, sport, and leisure-time (in this study, only the leisure-time domain was used). The leisure-time domain includes regular engagement in leisure-time activities, such as walking, cycling, watching television, and active transportation.

The frequency of cycling and walking (never, seldom, sometimes, often, and always) was assessed in all time points of the longitudinal study (baseline, 6-month, 12-month, and 18-month) and two new variables with score ranging from 4 to 20 points were created considering the sum of all responses. To categorize the sample according to the engagement in cycling and walking, participants who answered “never” in all four time points were classified as not engaged (cycling-no and walking-no, respectively) and those participants who reported any level of engagement during the 18 months were classified as engaged (cycling-yes and walking-yes, respectively).

### Covariates

General information of each participant included chronological age (difference between birthday and measurement day), sex (male and female), economic condition and formal education (standardized questionnaire) ^23^, and blood pressure (systolic - SBP, and diastolic blood pressure - DBP; measured by Omron HEM-742 model; https://www.omron-oficial.com/). Moreover, the previous diagnosis of chronic disease was registered at baseline (diabetes mellitus, arterial hypertension, and dyslipidemia).

### Statistical analysis

Descriptive statistics comprised mean, SD, and 95% confidence intervals (95%CI). Student’s t-test for independent samples compared participants engaged and not engaged in cycling and walking on leisure-time (Table 1). Spearman correlation assessed the relationship between healthcare costs accumulated during the 18 months and changes in physical activity, body composition, and blood pressure, as well as the stability of walking and cycling over time (Table 2). Analysis of variance (ANOVA) for repeated measures compared the impact of cycling and walking on body composition outcomes and healthcare costs (Tables 3 and 4, respectively). Sex, chronological age, economic condition score, and number of chronic disease were used as covariates in the ANOVA for repeated measures. Bonferroni’s post-hoc test was applied, when necessary, whereas measures of effect-size were expressed as eta-squared (ES-r). Statistical significance (p-value) was set at < 0.05 and the statistical software BioEstat, version 5.0 (https://mamiraua.org.br/downloads/programas/), ran all analyses.

**Table 1:** General characteristics of the sample stratified according to walking and cycling on leisure-time (n = 198).

Parameter	Cycling-no (n = 171)	Cycling-yes (n = 27)	Walking-no (n = 35)	Walking-yes (n = 163)
	Mean (SD)	Mean (SD)	Mean (SD)	Mean (SD)
Age (years) baseline	61.8 (8.7)	60.1 (8.7)	63.9 (9.8)	61.1 (8.4)
Body weight (kg) baseline	73.1 (15.2)	71.8 (9.4)	71.7 (14.4)	73.2 (14.6)
BMI (kg/m^2^)	29.8 (5.6)	27.2 (2.5) *	29.9 (5.5)	29.4 (5.3)
%BF-BIA baseline	40.1 (8.1)	33.2 (4.8) *	40.3 (8.9)	38.8 (7.8)
WC (cm) baseline	92.9 (13.8)	92.8 (7.8)	90.9 (10.9)	93.3 (13.6)
SBP (mmHg) baseline	130.6 (20.1)	126.3 (15.7)	132.6 (19.4)	129.4 (19.5)
DBP (mmHg) baseline	76.3 (11.4)	78.1 (8.1)	76.9 (8.8)	76.5 (11.5)
Economic condition score baseline	19.2 (4.7)	21.2 (4.7) *	19.2 (4.6)	19.5 (4.8)
Healthcare costs (USD) 24 months	369.19 (224.42)	274.36 (177.04) *	347.67 (177.50)	358.11 (229.23)
Arterial hypertension (yes) baseline [%]	67.3	37.1 *	68.6	62.1
Dyslipidemia (yes) baseline [%]	37.4	33.3	37.1	36.8
Diabetes mellitus (yes) baseline [%]	24.6	22.2	20.1	25.2

%BF-BIA: body fatness percentage; DBP: diastolic blood pressure; SBP: systolic blood pressure; SD: standard deviation; WC: waist circumference.

* p-value < 0.05 (yes versus no).

**Table 2:** Frequency of cycling and walking over the follow-up period (n = 198).

Frequency	Baseline	6-month	12-month	18-month	Spearman correlation (rho)
	n (%)	n (%)	n (%)	n (%)	
Cycling					
Never	180 (90.9)	185 (93.4)	183 (92.4)	183 (92.4)	Baseline × 6-month = 0.561 *
Seldom	5 (2.5)	3 (1.5)	7 (3.5)	6 (3.0)	Baseline × 12-month = 0.568 *
Sometimes	9 (4.5)	6 (3.0)	6 (3.0)	4 (2.0)	Baseline × 18-month = 0.586 *
Often	2 (1.0)	0 (0.0)	0 (0.0)	4 (2.0)	6-month × 12-month = 0.641 */6-month × 18-month = 0.630 *
Always	2 (1.0)	4 (2.0)	2 (1.0)	1 (0.5)	12-month × 18-month = 0.583 *
Walking					
Never	106 (53.5)	108 (54.5)	104 (52.5)	117 (59.1)	Baseline × 6-month = 0.349 *
Seldom	27 (13.6)	17 (8.6)	24 (12.1)	19 (9.6)	Baseline × 12-month = 0.225 *
Sometimes	25 (12.6)	26 (13.1)	21 (10.6)	24 (12.1)	Baseline × 18-month = 0.274 *
Often	13 (6.6)	19 (9.6)	28 (14.1)	19 (9.6)	6-month × 12-month = 0.342 */6-month × 18-month = 0.252 *
Always	27 (13.6)	28 (14.1)	21 (10.6)	19 (9.6)	12-month × 18-month = 0.288 *

* p-value < 0.01.

**Table 3:** Changes in body fatness markers and healthcare costs over 18 months follow-up according to cycling on leisure-time (n = 198).

	Baseline	6-month	12-month	18-month	ANOVA repeated measures (p-value) *		
	Mean (95%CI)	Mean (95%CI)	Mean (95%CI)	Mean (95%CI)	Time	Cycling	Time × Cycling
Costs (USD)					0.601	0.507	0.178
Cycling-no (n = 171)	83.97 (75.71-92.32)	87.63 (78.91-97.70)	92.98 (83.32-102.27)	95.42 (85.10-105.08)			
Cycling-yes (n = 27)	94.48 (72.62-116.34)	70.93 (47.94-93.82)	80.78 (55.45-105.08)	85.47 (58.26-112.59)			
WC (cm)					0.602	0.293	0.725
Cycling-no (n = 171)	93.1 (91.2-95.1)	96.8 (95.1-98.5)	96.2 (94.4-97.9)	96.7 (94.9-98.5)			
Cycling-yes (n = 27)	91.7 (86.6-96.7)	94.4 (89.7-99.1)	92.8 (88.3-97.4)	93.9 (89.3-98.6)			
%BF-BIA					0.304	0.028	0.973
Cycling-no (n = 171)	39.4 (38.3-40.4)	39.4 (38.4-40.5)	39.1 (37.9-40.1)	39.1 (37.9-40.1)			
Cycling-yes (n = 27)	36.1 (33.2-38.8)	36.3 (33.6-39.1)	35.8 (33.1-38.6)	35.7 (32.8-38.6)			

%BF: body fatness percentage; 95%CI: 95% confidence interval; ANOVA: analysis of variance; BIA: bioelectrical impedance analysis; WC: waist circumference.

* Model adjusted by sex, age, economic condition, and number of chronic diseases.

**Table 4:** Changes in body fatness markers and healthcare costs over 18 months follow-up according to walking on leisure-time (n = 98).

	Baseline	6-month	12-month	18-month	ANOVA repeated measures (p-value) *		
	Mean (95%CI)	Mean (95%CI)	Mean (95%CI)	Mean (95%CI)	Time	Cycling	Time × Cycling
Costs (USD)					0.452	0.562	0.608
Walking-no (n = 35)	86.04 (67.65-104.15)	84.53 (65.02-104.15)	81.25 (59.86-102.27)	86.51 (63.61-108.84)			
Walking-yes (n = 163)	85.29 (76.84-93.82)	85.57 (76.56-93.82)	93.35 (83.60-102.27)	95.79 (85.19-106.02)			
WC (cm)					0.395	0.799	0.202
Walking-no (n = 35)	91.5 (87.1-95.8)	97.4 (93.4-101)	95.7 (91.8-99.6)	95.1 (90.9-99.1)			
Walking-yes (n = 163)	93.2 (91.2-95.2)	96.2 (94.4-98.1)	95.7 (93.9-97.5)	96.6 (94.7-98.4)			
%BF-BIA					0.335	0.815	0.506
Walking-no (n = 35)	39.4 (37.1-41.8)	38.8 (36.5-41.1)	38.9 (36.6-41.3)	38.8 (36.3-41.3)			
Walking-yes (n = 163)	38.8 (37.2-39.9)	39.1 (37.9-40.1)	38.4 (37.3-39.5)	38.5 (37.3-39.7)			

%BF: body fatness percentage; 95%CI: 95% confidence interval; ANOVA: analysis of variance; BIA: bioelectrical impedance analysis; WC: waist circumference.

* Model adjusted by sex, age, economic condition, and number of chronic diseases.

## Results

The 198 participants that completed the study included more women than men (50 men [29.3%] and 148 women [70.7%]; p-value = 0.001), and the overall healthcare costs accumulated was USD 22,092.68. Men were older (p-value = 0.002), heavier (p-value = 0.006), taller (p-value = 0.001), and presented higher waist circumference (WC; p-value = 0.002) than women; they also had lower values %BF-BIA (p-value = 0.001) than women. Women were less engaged in cycling (p-value = 0.006) than men, and higher healthcare costs were observed among women (p-value = 0.029).

Participants engaged in cycling on leisure-time during the follow-up period had lower body fatness (p-value = 0.001), BMI (p-value = 0.001), healthcare costs (p-value = 0.038), and prevalence of arterial hypertension (p-value = 0.005) than participants not engaged in cycling. There was no significant difference according to the engagement in walking on leisure-time (Table 1).

Healthcare costs were inversely related to the 18-month cycling score (Spearman correlation - rho = -0.164; p-value = 0.021), but not the walking score (rho = -0.089; p-value = 0.215). Moreover, changes in WC (p-value = 0.210), %BF-BIA (p-value = 0.853), SBP (p-value = 0.301), and DBP (p-value = 0.953) were also unrelated to healthcare costs accumulated during the follow-up period.

In our sample, 86.4% (n = 171) of all participants reported “never” cycling in all four interviews, whereas 17.7% (n = 5) reported “never” walking in all four interviews. Prevalence of cycling was lower when compared with walking, and trends of the maintenance of walking over time were weak (rho ranging from 0.225 to 0.349) and for cycling were moderate (rho ranging from 0.561 to 0.641) (Table 2).

Over the follow-up period, participants who were more engaged in cycling presented lower %BF-BIA values (p-value = 0.028; cycling explained 2.7% of the variance observed on %BF-BIA [ES-r = 0.027]), but not WC (p-value = 0.293) (Table 3). In the crude analysis, participants with higher engagement in cycling presented lower healthcare costs over the follow-up period than those participants not engaged in cycling (p-value = 0.038; explaining 2.2% of all changes over time [ES-r = 0.022]). However, in the multivariate model, the impact of cycling was no longer significant (p-value = 0.507 [ES-r = 0.002]) mainly due to the impact of number of chronic disease (p-value = 0.001; ES-r = 0.194 [19.4%]).

Over the follow-up period, the engagement in walking did not significantly change WC (p-value = 0.799), %BF-BIA (p-value = 0.815), and healthcare costs (p-value = 0.562) (Table 4).

## Discussion

The 18-month longitudinal study involving adults showed that higher engagement in cycling on leisure-time affected body fatness levels, whereas its impact on economic parameters was explained by its role in the control of chronic diseas. Adopting a simple practice of physical activity during leisure-time seems relevant to prevent obesity and chronic disease, leading to a potential mitigation of healthcare costs.

In this study, men presented higher engagement in cycling than women, whereas walking was similar for the two genders. Regarding cycling, our findings are similar to other studies, describing that men have higher levels of leisure-time physical activity and use more cycling as transport than women ^16^ ^,^ ^24^. Regarding walking, the similar results for men and women also agree with previous data ^8^ and might be supported by walking being a cheaper physical activity, and its practice being spread out among adult population regardless of sex.

Another sex-dependent finding in our study was the higher healthcare costs among women. Cultural and biological aspects might support these differences in healthcare costs. Since early adolescence, girls are used to visit healthcare professionals to monitor aspects linked to birth control, which increases the use of primary care services and its costs. Moreover, menopause and its complications require close medical monitoring. On the other hand, men usually do not seek medical monitoring since culturally this act represents a demonstration of physical weakness, interfering in daily life activities that characterize their role as men in the society, such as labor activity ^25^.

In general, participants with higher cycling engagement during the follow-up had lower adiposity, agreeing with previous studies that have assessed the relationship between cycling and weight gain ^7^ ^,^ ^26^ ^,^ ^27^. Bassett et al. ^7^ assessed the prevalence of obesity, walking, and cycling in the United States, Europe, and Australia, describing that obesity prevalence was lower in geographical regions where active transport was more commonly reported by the participants. Moreover, among English workers aged 40-69 years-old (72,999 men and 82,788 women), those who commute using cycling and walking had lower average values for BMI and body fatness than those commuting by car ^26^. In fact, a reasonable explanation for this negative relationship might be the higher energy expenditure required to sustain physical activities such as cycling ^28^ ^,^ ^29^, leading to adiposity reduction when these activities are maintained for long periods ^29^.

Previous cross-sectional studies have identified a significant association between leisure-time physical activity and economic variables ^8^ ^,^ ^18^ ^,^ ^30^, but few addressed cycling. Cycling decreases the mortality risk by about 10% ^31^ and thus it may mitigate healthcare costs ^32^ ^,^ ^33^. In England and Wales, the promotion of higher engagement in walking and cycling would have the potential to save GBP 17 billion related to the prevention of chronic disease and reduction in the release of greenhouse gases in the atmosphere ^34^. Moreover, a cost-benefit analysis conducted in the United States indicated that each USD 2.79 spent building cycling paths would generate a gain of 0.0022 quality-adjusted life year ^35^.

In the crude analysis, the engagement in cycling on leisure-time affected healthcare costs by about 2%. In terms of magnitude, our findings are like other data from Canada and Brazil, ranging from 1% to 2.5% ^30^ ^,^ ^36^. On the other hand, multivariate models found that the association between cycling and lower chronic disease occurrence is the main contributor to the mitigation of healthcare costs. In fact, at baseline, participants engaged in cycling had a lower prevalence of arterial hypertension and this characteristic might affect its impact on costs during the follow-up period.

In terms of limitation, note that healthcare costs were based only in primary care services and, thus, secondary/tertiary services (e.g., surgeries, complex medical procedures), as well as productivity loss were not considered (e.g., absenteeism, presentism, and retirements due to health problems) ^37^. Second, the Likert scale used to describe the engagement in cycling and walking does not represent measures of both frequency and volume, limiting the exploratory analysis linking both behaviors to the outcomes assessed in our study. Third, the minimum sample size required to the statistical analysis (n = 75) was not reached in the group of participants engaged in cycling activities, limiting the power of our conclusions. Finally, cycling and walking are behaviors manifested in other physical activity domains (e.g., active transportation, labor activities), thus not monitoring these behaviors in other domains is a limitation of our study.

## Conclusion

In summary, the findings of this longitudinal study hint that cycling on leisure-time is inversely related to adiposity in adults and that the role of cycling on chronic disease prevention seems to be the main pathway linking it to the mitigation of healthcare costs.
